# Announcement of the Registered Report "Effect of Cultural Adaptation of a Smartphone-Based Self-Help Programme on its Acceptability and Efficacy"

**DOI:** 10.32872/cpe.v2i3.4281

**Published:** 2020-09-30

**Authors:** Eva Heim, Sebastian Burchert, Mirëlinda Shala, Marco Kaufmann, Arlinda Cerga Pashoja, Naser Morina, Michael P. Schaub, Christine Knaevelsrud, Andreas Maercker

**Affiliations:** a Department of Psychology, University of Zurich, Zurich, Switzerland; b Department of Education and Psychology, Freie Universität Berlin, Berlin, Germany; c Epidemiology, Biostatistics and Prevention Institute, University of Zurich, Zurich, Switzerland; d Faculty of Population Health, London School of Hygiene and Tropical Medicine, London, United Kingdom; e Public Health England, London, United Kingdom; f Department of Psychiatry and Psychotherapy, University Hospital Zurich, Zurich, Switzerland; g Swiss Research Institute for Public Health and Addiction, Zurich, Switzerland; Philipps-University of Marburg, Marburg, Germany

In order to narrow the world-wide treatment gap, innovative interventions are needed that can be used among culturally diverse groups, e.g., immigrant populations in high-income countries. Research on cultural adaptation of psychological interventions indicates that a higher level of adaptation is associated with a higher effect size of the intervention. However, direct comparisons of different levels of adaptations are scarce and have not been done with self-help interventions.

## Aims

The registered study will use a Smartphone-based self-help programme called Step-by-Step (Albanian: Hap-pas-Hapi) for the treatment of psychological distress among Albanian-speaking immigrants in Switzerland and Germany. Two levels of cultural adaptation (i.e., surface vs. deep structure adaptation) will be compared. We hypothesise that the deep structure adaptation will enhance the acceptance and effect size of the intervention. The deep structure adaptation was done based on an ethnopsychological study to examine the target population’s cultural concepts of distress.

## Method

In the registered study, we will conduct a two-arm, single-blind randomised controlled trial. Participants will be randomly assigned to the surface vs. deep structure adaptation version of Hap-pas-Hapi (1:1 allocation using permuted block randomization). Inclusion criteria are good command of the Albanian language, age above 18, and elevated psychological distress (Kessler Psychological Distress Scale score above 15). Primary outcome measures are the total score of the Hopkins Symptom Checklist and the number of participants who completed at least three (out of five) sessions. Secondary outcomes are global functioning, well-being, symptoms of post-traumatic stress, and self-defined problems. In addition, we will test a mediation model, hypothesizing that the deep structure adaptation will address fatalistic beliefs and enhance alliance with the self-help programme, which in turn increases the acceptance and effect size of the intervention. And finally, we will measure acculturation and hypothesise, that with higher levels of acculturation, the effect of the deep structure adaptation will diminish.

## Discussion

The registered study is the first study to directly compare two different levels of cultural adaptation of an online self-help programme for the treatment of psychological distress among immigrants in high-income countries. We aim to deliver theory-driven and methodologically rigorous empirical evidence regarding the effect of cultural adaptation on the acceptance and effect size of this self-help programme.

## Supplementary Materials

The study protocol for this Registered Report is publicly accessible via PsychArchives.org (see Index of Supplementary Materials below).



HeimE.
BurchertS.
ShalaM.
KaufmannM.
Cerga PashojaA.
MorinaN.
SchaubM. P.
KnaevelsrudC.
MaerckerA.
 (2020). Effect of cultural adaptation of a smartphone-based self-help programme on its acceptability and efficacy: Study protocol for a randomized controlled trial. PsychOpen. 10.23668/psycharchives.3152PMC1130391739119053

## Notes

**Editor's note:** This is an announcement of a Registered Report which received In-Principal-Acceptance (IPA) to be published in "Clinical Psychology in Europe". The study protocol is publicly accessible at https://doi.org/10.23668/psycharchives.3152. In this announcement, a brief summary of the study protocol is presented.
